# Early-life interventions to prevent feather pecking and reduce fearfulness in laying hens

**DOI:** 10.1016/j.psj.2023.102801

**Published:** 2023-05-24

**Authors:** Saskia Kliphuis, Maëva W.E. Manet, Vivian C. Goerlich, Rebecca E. Nordquist, Hans Vernooij, Henry van den Brand, Frank A.M. Tuyttens, T. Bas Rodenburg

**Affiliations:** ⁎ Animals in Science and Society, Department of Population Health Sciences, Faculty of Veterinary Medicine, Utrecht University, Utrecht, the Netherlands; † Adaptation Physiology Group, Department of Animal Sciences, Wageningen University and Research, Wageningen, the Netherlands; ‡ Flanders Research Institute for Agriculture, Fisheries and Food (ILVO), Merelbeke, Belgium; § Department of Veterinary and Biosciences, Faculty of Veterinary Medicine, Ghent University, Merelbeke, Belgium

**Keywords:** pullet, chicken, prenatal, maladaptive behavior, stress

## Abstract

Severe feather pecking, the pulling out of feathers of conspecifics, is a major welfare issue in laying hens. Possible underlying causes are fearfulness and lack of foraging opportunities. Because early life is a crucial stage in behavioral development, adapting the incubation and rearing environment to the birds' needs may reduce fearfulness and prevent the development of feather pecking. In a 2 × 2 factorial design study, we investigated whether a green light-dark cycle throughout incubation, which resembles natural incubation circumstances more than the standard dark incubation, and foraging enrichment with live larvae during rearing reduce fearfulness and feather pecking and increase foraging behavior of laying hen pullets from an early age onwards. In this 2-batch experiment, 1,100 ISA Brown eggs were incubated under either 0 h of light/24 h of darkness or 12 h of green LED light/12 h of darkness. After hatching, 400 female chicks (200 per batch) were housed in 44 pens (8–10 chicks per pen). During the entire rearing phase (0–17 wk of age), half of the pens received black soldier fly larvae in a food puzzle as foraging enrichment. We assessed fear of novel objects and humans, feather pecking, plumage condition, foraging behavior, and recovery time after a 3-fold vaccination (acute stressor). A slight increase in the number of foraging bouts was only seen with larvae provisioning (rate ratio 1.19, 95% CI 1.02–1.29, *P* = 0.008). Neither lighted incubation nor larvae provisioning affected fearfulness, feather pecking, plumage condition or recovery time after vaccination. In conclusion, the present study showed no effects of light during incubation and minor effects of foraging enrichment during rearing on the behavior of laying hen pullets. Further research is recommended on other welfare aspects.

## INTRODUCTION

Worldwide, over 7 billion laying hens are kept annually for egg production (Fernyhough et al., 2020). These hens are often kept under conditions that prevent opportunities to express species-specific behaviors like foraging. This lack of behavioral opportunities can lead to welfare problems and maladaptive behavior, such as feather pecking (**FP**). FP is defined as the pecking at and pulling out of feathers of conspecifics, leading to pain, baldness, injuries, and even mortality. This behavior is, therefore, a major welfare issue in the poultry industry. FP has multiple underlying causes, including fearfulness and lack of foraging opportunities (Rodenburg et al., 2013).

Commercial poultry practice in the European Union (**EU**) has been to routinely beak-trim laying hen chicks, that is, remove the tip of the beak to prevent damage caused by FP. Due to societal and ethical concerns, however, this procedure is now banned in the Netherlands and several other EU countries. It has been prohibited in organic husbandry since 2008 (EC No 889/2008). Because of the ban, the risk of FP outbreaks is again a problem and keeping hens with intact beaks can result in more severe damage than keeping flocks with trimmed beaks (Schwarzer et al., 2021). Consequently, innovative solutions need to be found to prevent or reduce the development of FP in laying hens.

The environmental conditions during incubation play a major role in the development of stress responsivity in chicks. Experiences during the incubation period are also known to affect subsequent sensitivity to stressors and stimuli in later life (Goerlich et al., 2012; Janczak and Riber, 2015). Eggs in most commercial hatcheries are incubated in complete darkness. However, under natural conditions, the mother hen would occasionally leave the nest during the last days of incubation in search of food, during which her eggs are periodically, though shortly, exposed to daylight (Mrosovsky and Sherry, 1980; Archer and Mench, 2014a). Due to the embryo's position in the egg at the end of incubation, the right eye is exposed to light from outside the eggshell (Koshiba et al., 2003). This asymmetrical light exposure plays a major role in modulating lateralization of the avian brain; light stimulates the right-eye system (i.e., the right eye and left brain hemisphere), leading to a more dominant left hemisphere (Rogers, 2008). Multiple studies have demonstrated that birds with a dominant left hemisphere are less sensitive to stressors, because they are better able to control the fear response initiated by the right hemisphere (reviewed in Rogers, 2010). Light exposure throughout incubation has also been shown to affect fear-related behavior and FP (Riedstra and Groothuis, 2004; Archer and Mench, 2014b; Chiandetti and Vallortigara, 2019). The majority of these studies, however, were performed with broilers not laying hens. Moreover, the existing body of literature was not consistent regarding the effect of lighted incubation on behavior, with some reporting a reduction in fearfulness (Archer and Mench, 2014a; Archer, 2017) and FP (Özkan et al., 2022) and others an increase in fearfulness (Dimond, 1968) and (gentle) FP (Riedstra and Groothuis, 2004). The light color used in these studies also seemed to play a key role in these reported effects, with green light demonstrating a decrease in FP (Özkan et al., 2022). The use of green light, therefore, seems promising as an intervention to promote the welfare of laying hens.

After hatching, the rearing environment continues to play an important role in the behavior development of laying hen pullets (Campbell et al., 2019; De Haas et al., 2021). Optimizing conditions in the rearing environment, therefore, might improve the welfare of laying hens throughout their entire lifespan. For example, a study by Bestman et al. (2009) showed that hens reared with natural light seemed less prone to develop FP than hens reared with artificial light. Furthermore, previous studies demonstrated that enrichment provided in the rearing environment can stimulate brain development and reduce fearfulness (De Haas et al., 2014b; Brantsæter et al., 2016; Campbell et al., 2019). Foraging enrichment, for example, providing black soldier fly larvae (**BSFL**), could potentially be used as effective environmental enrichment for laying hen pullets because insects are part of the natural diet of chickens and their movement is highly attractive to these birds. BSFL have a unique nutritional profile and they can be used efficiently in the sustainable breakdown of organic waste material (Khan, 2018). Since laying hens under natural conditions spend most of their waking hours pecking and scratching in their search for food, providing larvae in cage-free housing systems could stimulate the expression of foraging. Expressing such behavioral needs is most likely pleasurable (Widowski and Duncan, 2000). In addition, the provision of foraging enrichment during rearing could reduce the risk of developing FP in later life (Tahamtani et al., 2016). Previous studies have shown that enrichment with live BSFL had potential welfare benefits for broilers (Ipema et al., 2020) and adult laying hens (Star et al., 2020; Tahamtani et al., 2021). Furthermore, larvae provisioning did not affect—positively or negatively—laying hen performance (Ruhnke et al., 2018). Despite the potential, no studies have yet investigated the effects of live BSFL provided throughout the rearing phase on the behavior and development of FP in laying hen pullets.

In summary, optimizing incubation and rearing conditions of laying hen pullets may reduce fearfulness, promote the expression of behavioral needs, and prevent the development of FP. Research on the combined effects of incubation and environmental enrichment during rearing in these pullets is, however, lacking despite the high demand for solutions applicable to the poultry sector. To meet this demand and to develop management strategies that enhance the welfare of laying hens, we investigated whether the presence or absence of green LED light during incubation and foraging enrichment with BSFL during rearing affect fearfulness and FP in laying hen pullets. Our hypothesis was that laying hen pullets exposed to lighted incubation and provided with BSFL during rearing would be less fearful, would show the less FP, would have a better plumage condition, and would forage more compared with control pullets (dark incubation and no larvae enrichment).

## MATERIALS AND METHODS

### Ethical Statement

This experiment was approved by the Dutch Central Authority for Scientific Procedures on Animals (CCD) under license number AVD1080020198685, and by the Animal Welfare Body Utrecht under work protocol numbers 8685-1-01 and 8685-1-03. The study was executed in accordance with the Dutch legislation and the EU directive on animal experimentation.

### Experimental Design

This experiment was conducted in 2 batches and used a 2 × 2 factorial arrangement combining light vs dark incubation and presence vs absence of larvae during rearing, 4 treatments were defined: dark, no larvae [**DnL**]; dark, larvae [**DL]**; light, no larvae [**LnL**]; light, larvae [**LL**]. Chicks were randomized and housed in 44 pens (20 pens in batch 1; 24 pens in batch 2). Treatments were not mixed within pens. Due to a randomization error, the treatments were unequally divided over the pens in batch 1 (3:7:7:3 [DnL:LnL:DL:LL, order used throughout]). To compensate for this unequal sample size, 4 extra pens were built to increase statistical power in batch 2 (7:5:5:7). Since there were 200 laying hen pullets per batch, group sizes per pen differed between the batches (10 in batch 1, 8 or 9 in batch 2). For both batches combined, the total numbers of pens per treatment were 10:12:12:10. To avoid incubator effects, approximately equal numbers of chicks from incubators with the same light treatment were housed within a pen.

### Incubation Conditions

A total of 1,100 eggs of the layer hybrid ISA Brown (Hendrix Genetics, obtained from hatchery "Het Anker" in Ochten, NL) were incubated at the research facility of Wageningen University & Research in Wageningen, NL, in 2 batches (500 eggs in batch 1 [January 2020]; 600 eggs in batch 2 [April 2021]). Logistically it was not possible to perform both batches in the same season, because the research facility was unavailable in that period. Age of the parent stock was 43 wk and 34 wk, respectively. In each batch, eggs were randomly assigned to 1 of 2 conditions: Light or Dark incubation. The Light eggs were exposed to 12 h of green LED light and 12 h of darkness per day, whereas the Dark eggs were not exposed to any light during incubation. There were 2 incubators per treatment; 1 HatchTech incubator with an egg set capacity of 1,400 eggs, and 1 climate respiration chamber with an egg set capacity of 400 eggs. For more information about the incubators, see Güz et al. (2021). The light treatment was switched between incubators between consecutive batches. Light originated from monochrome green LED strips (Barthelme Y51515213 182007 LED strip, 520 nm), attached directly above each tray containing eggs, providing 400 lux at egg level. The eggs were turned automatically 90° every hour. Eggshell temperature was set to 37.8 ± 0.2°C on E0 (embryonic d 0) and decreased to 36.2°C on E18 and to 35.7°C on E20. Relative humidity was set to 57.5% on E0 and to 58.5% on E18. On E18, the eggs were candled and those containing a vital embryo were transferred to hatching baskets. After hatching, the chicks were color-sexed; female chicks were then health-checked (protocol described in Heijmans et al., 2022) and given a neck label for individual identification. Male chicks and surplus female chicks were culled by cervical dislocation. The chicks were not beak-trimmed. At 1 d of age, 200 female chicks per batch were transported to Utrecht, NL, where they were housed at the Farm Animal Health research facility in the faculty of Veterinary Medicine at Utrecht University throughout the rearing phase, until 17 wk of age.

### Rearing Conditions

The rearing pens were each 246×88×241 (l×w×h) cm, separated by wire mesh and a 60-cm high wooden barrier to prevent visual contact between adjacent pens. The pen floors were initially covered with peat as litter material to create a larger visual contrast with the larvae. The peat was replaced with wood shavings from wk 8 onwards after the occurrence of severe eye infections in 2 chicks (see Batch effects discussion in Supplementary data). A ceramic heat lamp, perches, a water bucket with 3 drinking nipples, and a round feeder were provided in each pen. Light was provided via vertical high-frequency dimmable bird lights (GlassLux Standard 1 × 36W Philips, Eindhoven, NL) and daylight entered the poultry house through skylights with automated hatches (Boon Agrosystems, Barneveld, NL). In this way, the hours of daylight could be controlled as needed. The number of light hours was decreased from 23 h at 1 d of age to 12 h at 5 wk of age, where it remained until the end of the study. Since the experimental batches were conducted in different seasons (Feb-June 2020 and May-Sep 2021), daylight control allowed standardization of the light-dark cycle during rearing. Heat lamps provided a temperature of 35°C at floor level at 1 d of age and were adjusted in height above the floor over the following days, thus lowering the temperature in the pens. Room temperature was gradually lowered from 25°C at 1 d of age to 18°C at 5 wk of age and remained constant thereafter. A radio played classical music in the poultry house 24/7 throughout the experiment to avoid strong responses to environmental noise and entering humans (Davila et al., 2011). Pullets received vaccinations according to a standard Dutch/Belgian vaccination scheme. The vaccination scheme was in accordance with the Belgian legislation for commercial egg production because the pullets would be transported to the Flanders Research Institute for Agriculture, Fisheries and Food (ILVO) in Melle, BE, for follow-up research at 19 wk of age. In the first week of life, the pullets received a commercially available rearing diet (Starter 1, De Heus, Ede, NL). After that, a new diet was gradually mixed in (see next paragraph).

### Larvae Enrichment

From 7 d of age onwards, chicks in half of the pens received live BSFL (batch 1: from Circular Organics; batch 2: first from Circular Organics [Turnhout, BE], then from Bestico [Berkel en Rodenrijs, NL] from 6 wk of age onwards, due to bankruptcy of Circular Organics). The number of larvae provided corresponded with 10% of the daily feed intake as described in the ISA Brown product guide, and therefore increased with age. Tailor-made diets (Research Diet Services, Wijk bij Duurstede, NL) gradually replaced the commercially available diet from 7 d of age onwards. This diet included additional protein and BSF oil in the diet of the no-larvae pullets (DnL, LnL), to compensate for any nutritional effects caused by the 10% larvae feeding in the DL and LL groups. The larvae were provided in transparent cylinders (15 × 4 cm) containing three 9-mm holes each. This design was based on a previous study on broilers (Ipema et al., 2020). A pilot study performed by the authors in December 2019 confirmed that this design was also suitable for laying hen pullets. Two dispensers with fresh larvae were put in each pen 6 d per week by caretakers during the daily checks, from 1 to 19 wk of age. On testing days, larvae tubes were always provided 1 h before testing started.

### Behavior Tests and Observations

All behavior tests described in the present study were performed at pen level. Plumage condition was scored per individual pullet. Observers were blind to the incubation treatment, but not to the larvae treatment, as larvae dispensers were present during the observations. Detailed test protocols, as well as the corresponding raw datafiles, are available in the DANS repository (DOI: 10.17026/dans-26r-bywc). A timeline of all measurements described in the present study is provided in Table 1. The pullets in this experiment were also subjected to an array of other tests and observations (see extended overview in supplementary data).

**Table 1:** Timeline of behavior tests and observations in batch 1 (Feb-Jun 2020) and batch 2 (May-Sep 2021).

Age (wk)	1	2	3	4	5	6	7	8	9	10	11	12	13	14	15	16	17
Batch 1	NO-1 Start larvae				FP					NO-2 HA					PC		
Batch 2	NO-1 FB-1 Start larvae			FB-2	FP		FB-3			NO-2 HA			BR		PC		

Abbreviations: BR, behavioral recovery after a stressor; FB, foraging behavior observations (3 ages); FP, feather pecking observations; HA, human approach test; NO, novel object test (2 ages); PC, plumage condition scoring.

NO-1 and FB-1 were performed before starting the larvae provisioning.

The COVID lockdown is illustrated in grey.

*Novel Object Test.* The response to a novel object (**NO**) was assessed at 6 d (NO1) and 10 wk (NO2) of age. After sitting in front of the pen for 5 min as habituation, the observer placed a NO in the middle of the pen floor. The latency for the first pullet to approach the object within a 3-chicken distance was scored. The test duration was 4 min. During NO1, the object was a rectangular-shaped piece of wood, covered with tape in multiple colors. During NO2, the object was a beverage can. The observation protocol was adapted from De Haas et al. (2014a). Since larvae provisioning had not yet started at NO1, only incubation effects were analyzed (N = 44).

*Human Approach Test*. Fear of humans was measured at 10 wk of age during a human approach test (HA). The observer stood stationary at the pen entrance for 5 min, and the latency of the first 3 pullets to approach was recorded (N = 44).

*Behavioral Recovery after a Stressor.* Behavioral recovery data were collected for the pullets in batch 2 only. At 13 wk of age, all pullets received 3 consecutive vaccinations, which were a wing web vaccination (fowl pox), an eye drop vaccination (infectious laryngotracheitis), and an intramuscular vaccination in the pectoral muscle (Newcastle disease, infectious bronchitis, egg drop syndrome, and turkey rhinotracheitis). To assess whether or not the treatments affected the recovery time after an acute stressor, chicken behavior was scored at pen level for 60 min after vaccination, using scan sampling with 10-min intervals (Table 2). The same observations were performed on d 9, 10, and 11 after the vaccination, without any disturbances. The mean number of pullets per behavior per time interval across these 3 undisturbed days was considered the baseline time budget. In the analysis, the time the pullets took to return to baseline, expressed as the time interval*behavior interaction, was considered to be an indicator of recovery time after a stressor. Behaviors were scored on video by 1 observer. The observer was blind to the incubation treatment, but not to the larvae treatment. Five pens were excluded due to errors in the recording system (N = 19, 5:4:4:6 pens per treatment). The protocol was adapted from Ericsson et al. (2014).

**Table 2:** Ethogram during behavioral recovery after a stressor (Ericsson et al., 2014).

Behavior	Description
Inattentive	Standing, sitting, or walking with reduced attention (i.e., no alert head movements and neck slightly retracted); eyes may be partially closed while standing and sitting
Alert	Standing, sitting, or walking with eyes opened and neck raised, attentive to the surroundings but not to floor, feed, or water bucket
Preening	Using beak to trim and arrange feathers
Foraging	Pecking and scratching the ground or larvae tube
Feeding	Feeding from yellow food container
Drinking	Drinking from the water bucket
Dust bathing	Squatting and tossing wood shavings onto body, followed by an organized sequence of behavior patterns such as head rubbing and vertical wing shaking
Feather ruffling	Erecting feathers and shaking body
Wing flapping	Flapping wings while standing on ground or perch

*Feather Pecking.* We scored FP at 5 wk of age, because early FP has been reported to occur at this age (De Haas et al., 2014a). Pecks were scored as bouts, defined as either 1 peck or a series of pecks with less than 1-s intervals. Gentle (**GFP**) and severe (**SFP**) feather pecks were defined according to Newberry et al. (2007) (Table 3). FP was scored continuously at 3 different times of day for 25 min each, after 5 min of habituation, by different observers per pen and time of day. The FP observation protocol was adapted from De Haas et al. (2014a). Five pens from batch 1 were excluded because a rooster was present due to sexing error. One pen in batch 1 was excluded from analysis due to a missed observation session (N = 38). Preceding data collection, a training session including live observations took place in which the same pen was scored until an inter-observer reliability with Kendall coefficient of 0.8 was reached.

**Table 3:** Ethogram for gentle (GFP) and severe (SFP) feather pecking (Newberry et al., 2007).

Behavior	Description
GFP	Bird makes gentle beak contact with the feathers of another bird without visibly altering the position of the feathers. Bird usually stands behind or to the side of the recipient, who makes no apparent response
SFP	Bird grips and pulls or tears vigorously at a feather of another bird with her beak, causing the feather to lift, break, or be pulled out. Bird usually stands behind or to the side of the recipient, who reacts to the peck by vocalizing, moving away, or turning towards the pecking bird

*Plumage Condition.* At 15 wk of age, plumage condition was scored according to Bilcik and Keeling (1999). Eleven body regions were assessed for broken or missing feathers and wounds, on a 0 to 5 scale (0 being intact feathers and 5 being completely denuded). A cumulative score from all body regions was calculated for each individual bird.

*Foraging Behavior.* Foraging behavior was observed at 1, 4, and 7 wk of age (see Table 2 for definition of foraging) and only in the batch 2 pullets (N = 24). One focal animal was followed for 5 min, during which foraging duration and the number of foraging bouts were noted. A foraging bout was considered finished when the pullet raised her head for 2 s, or when another behavior commenced. Six pullets per pen were observed, hence the total observation time per pen lasted 30 min at every age (90 min per pen in total).

### Statistical Analysis

Statistical analyses were performed in R version 4.1.2 (R Core Team, 2022). All outcome variables were analyzed at pen level, except for plumage condition, where the pen was included as a random factor. Dynamics (e.g., behavior) within a pen can be different in another, making pullets in the same pen more like each other than pullets in different pens. Batch was added to all models as fixed effect, given the systematic differences between the 2 batches (see supplementary data) which cannot be estimated robustly as random effect. The outcome variables for the behavioral recovery after vaccination were transformed to visualize the deviation from baseline behavior. In addition, the baseline values were incorporated in the model as an offset to correct for behavior on undisturbed days (data normalization). For the latency to approach, a parametric survival analysis with log-normal distribution was used, taking censoring into account in cases when data reached the cutoff point (Therneau, 2021). The log-normal distribution was selected from several other parametric distributions based on the Akaike information criterion. For count variables (e.g., number of GFPs), a generalized linear mixed model was used with Poisson or negative binomial distribution (Brooks et al., 2017), chosen after visual inspection of the variance, using the DHARMa package (Hartig, 2022). In all models, incubation condition (i.e., green light versus darkness) and larvae condition (i.e., larvae provisioning versus no larvae), and—if performed in both batches—batch were included in the full model as fixed factors, as was incubation*larvae interaction. A backward model selection procedure was followed, using Akaike information criterion to assess model fit improvement. Since the incubation*larvae interaction did not improve any of the models, it was always removed. Factors incubation and larvae condition remained in the model to assess the effect size. Effect sizes are reported as rate ratios (**RR**, ratio of mean number in condition *X*/mean number in reference condition) in case of count data and hazard ratios (**HR**) in case of latency data, both with 95% CI. A 95% CI covering value 1 (unity) means there was no difference between the means of the 2 conditions. Effects are considered significant at *P* ≤ 0.05. Plumage condition was analyzed with Bayesian multinomial logistic regression using Stan (Bürkner, 2017).

## RESULTS

An overview of the raw data mean outcomes of the behavioral tests and scoring of plumage condition is provided in Table 4.

**Table 4:** Means ± SEM of the raw data of outcome parameters for all treatment groups and total population.

Variable	DnL	LnL	DL	LL	Grand mean
*Novel object test*					
Approach latency (s)—6 d	139.7 ± 22.1	162.1 ± 18.5	NA	NA	120.3 ± 15.7
Approach latency (s)—10 wks	57.4 ± 10.0	62.5 ± 8.0	55.2 ± 18.8	93.9 ± 30.8	66.5 ± 9.2
*Human approach test*					
Approach latency (s)	95.3 ± 28.5	101.1 ± 26.5	106.3 ± 26.1	139.0 ± 24.3	109.8 ± 13.0
*Feather pecking*					
GFP (n)	26.4 ± 5.4	37.7 ± 9.9	33.7 ± 6.6	18.3 ± 2.4	29.4 ± 3.5
SFP (n)	2.8 ± 1.1	3.6 ± 1.3	4.2 ± 2.5	2.0 ± 1.0	3.2 ± 1.8
*Plumage condition*					
Cumulative plumage score^1^	1.3 ± 0.1	1.8 ± 0.1	1.4 ± 0.1	1.3 ± 0.1	1.5 ± 0.1
*Foraging*					
Foraging duration (s)	45.4 ± 4.1	41.3 ± 4.4	50.2 ± 5.2	56.6 ± 4.5	48.7 ± 2.3
Foraging bouts (n)^2^	**2.6 ± 0.2^a^**	**2.7 ± 0.2^a^**	**3.0 ± 0.2^b^**	**3.1 ± 0.2^b^**	2.8 ± 0.1

Abbreviations: DL, dark, larvae; DnL, dark, no larvae; GFP, gentle feather pecks; LL, light, larvae; LnL, light, no larvae; NA, data not collected because larvae provisioning started at 7 d of age; SFP, severe feather pecks.

1 Cumulative 0 to 5 score for 11 body regions, 0 = intact and 5 = completely denuded.

2 Means with a different superscript are significantly different (*P* ≤ 0.05).

*Fear Tests (NO and HA Tests).* Light during incubation did not affect latency to approach the NO at 6 d of age (HR 1.50, 95% CI 0.85–2.62; *P* = 0.16). Similarly, the NO test at 10 wk of age showed no effects of light during incubation (HR 1.12, 95% CI 0.49–2.60; *P* = 0.79) or larvae provisioning (HR 0.63, 95% CI 0.27–1.42; *P* = 0.26). The latency to approach a familiar human in the HA test was not affected by either light during incubation (HR 1.56, 95% CI 0.74–3.31; *P* = 0.27) or larvae provisioning (HR 1.20, 95% CI 0.55–2.57; *P* = 0.69).

**Figure 1:**
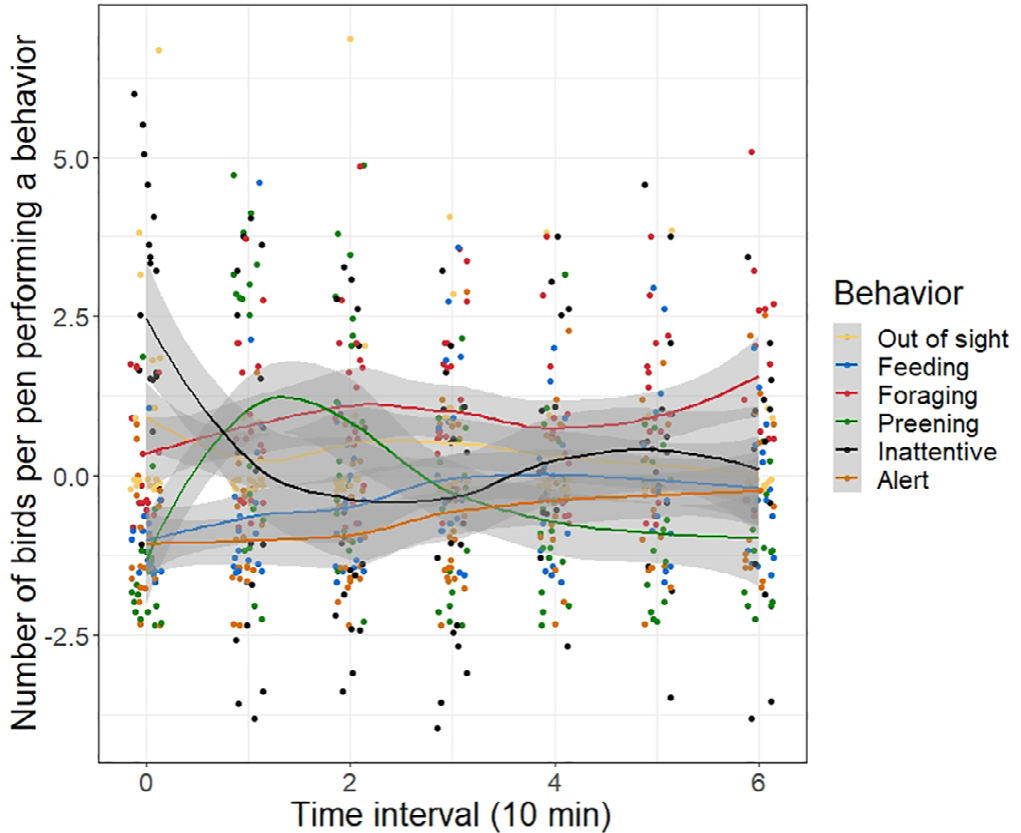
Dot plot with smoothed lines of the mean number of pullets per behavior for each time interval after vaccination in laying hen pullets at 13 wk of age. Baseline count was taken into account, calculated as the mean number of pullets performing the behavior during 60 min on the 3 undisturbed observation days. Y = 0 represents the baseline. Every dot is a cumulated number at pen level.

*Behavioral Recovery after a Stressor.* Light during incubation and larvae provisioning did not affect behavior in the 60 min after the 3-fold vaccination compared with behavior on the baseline days (*P* > 0.05). Irrespective of treatment, preening behavior increased immediately after vaccination (RR 3.62, 95% CI 2.01–7.09; *P* < 0.001; Figure 1).

**Figure 2:**
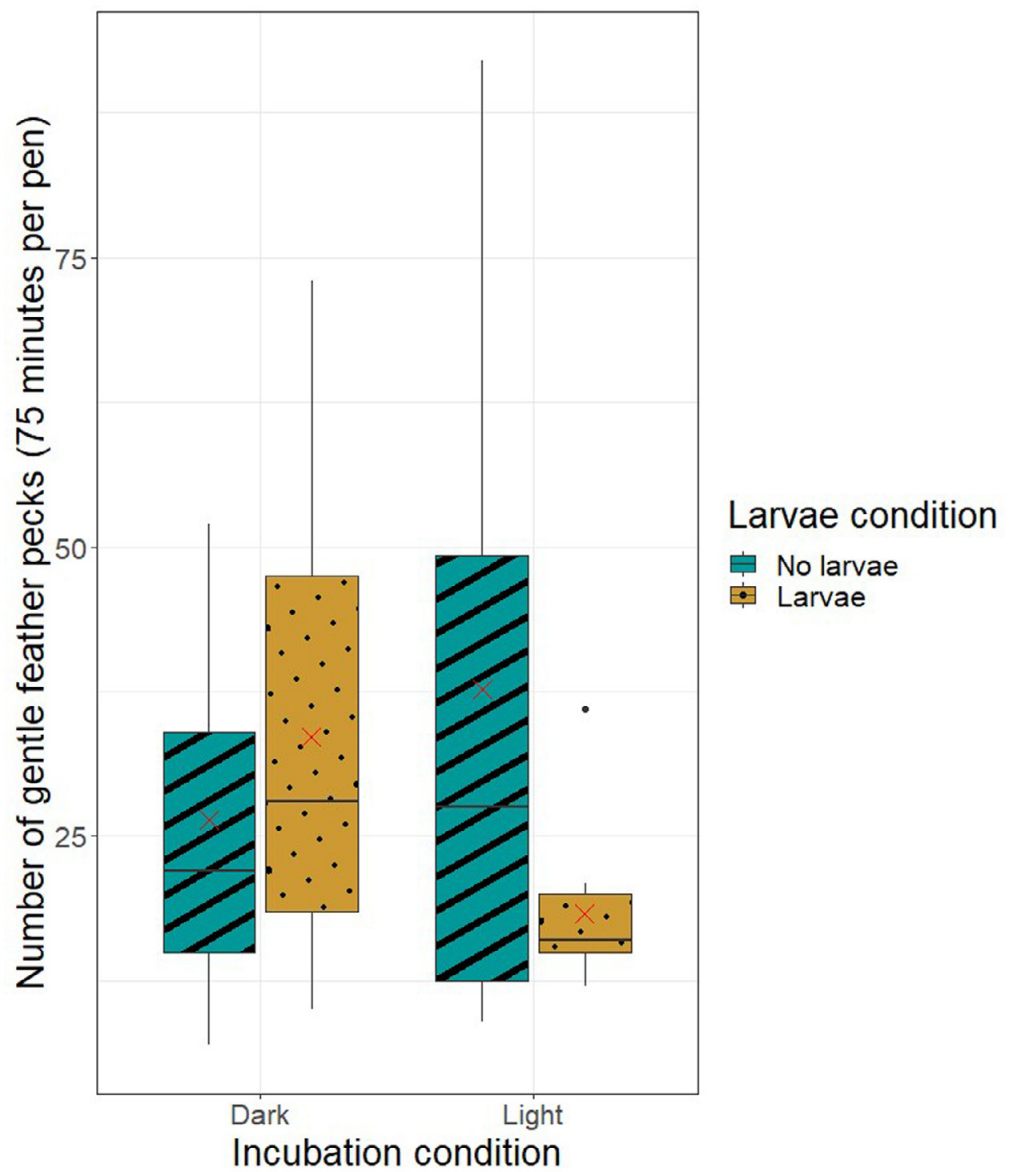
Boxplot of the total number of gentle feather pecks (GFP) per treatment throughout the 75-min observation time at 5 wk of age. The X in the box represents the mean.

*Feather Pecking.* Neither light during incubation (RR 0.87, 95% CI 0.69–1.11; *P* = 0.97) nor larvae provisioning during rearing (RR 0.81, 95% CI 0.63–1.02; *P* = 0.68) affected the number of GFPs (Figure 2). The numbers of SFPs observed were too few to analyze.

**Figure 3:**
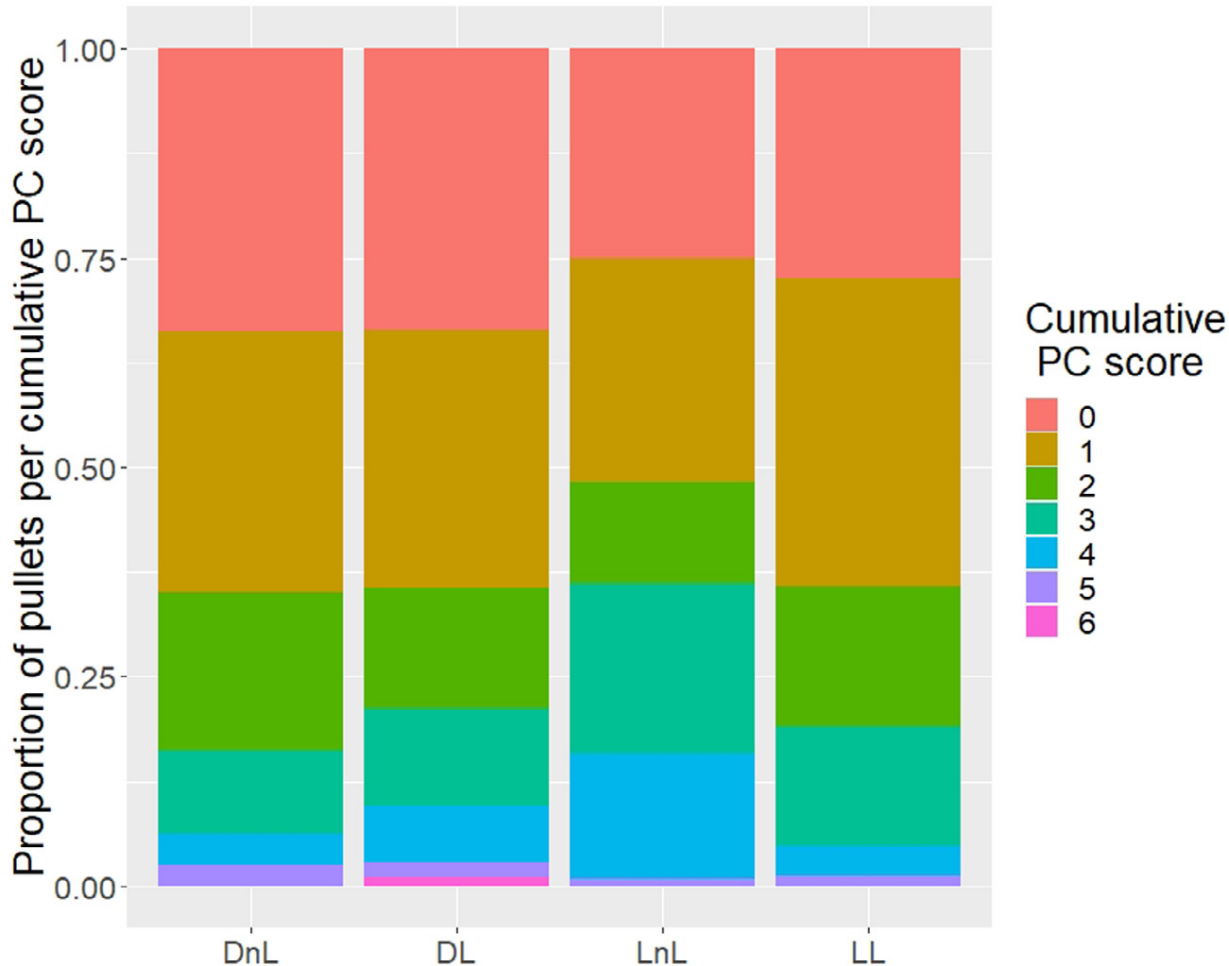
Stacked bar plot of the proportion of pullets with a given cumulative plumage condition (PC) score at 15 wk of age in each treatment group. Eleven body regions were given a score on a 0 to 5 scale, with 0 being intact feathers and 5 being completely denuded (Bilcik & Keeling, 1999). The scores of these body regions are added together to form the cumulative PC score. Abbreviations: DL, dark, larvae; DnL, dark, no larvae; LL, light, larvae; LnL, light, no larvae.

*Plumage Condition.* No effects of light during incubation (OR 1.53, 95% CI 0.75–3.10) or larvae provisioning (OR 0.76, 95% CI 0.7–1.53. Figure 3) were found regarding cumulative plumage condition score.

**Figure 4:**
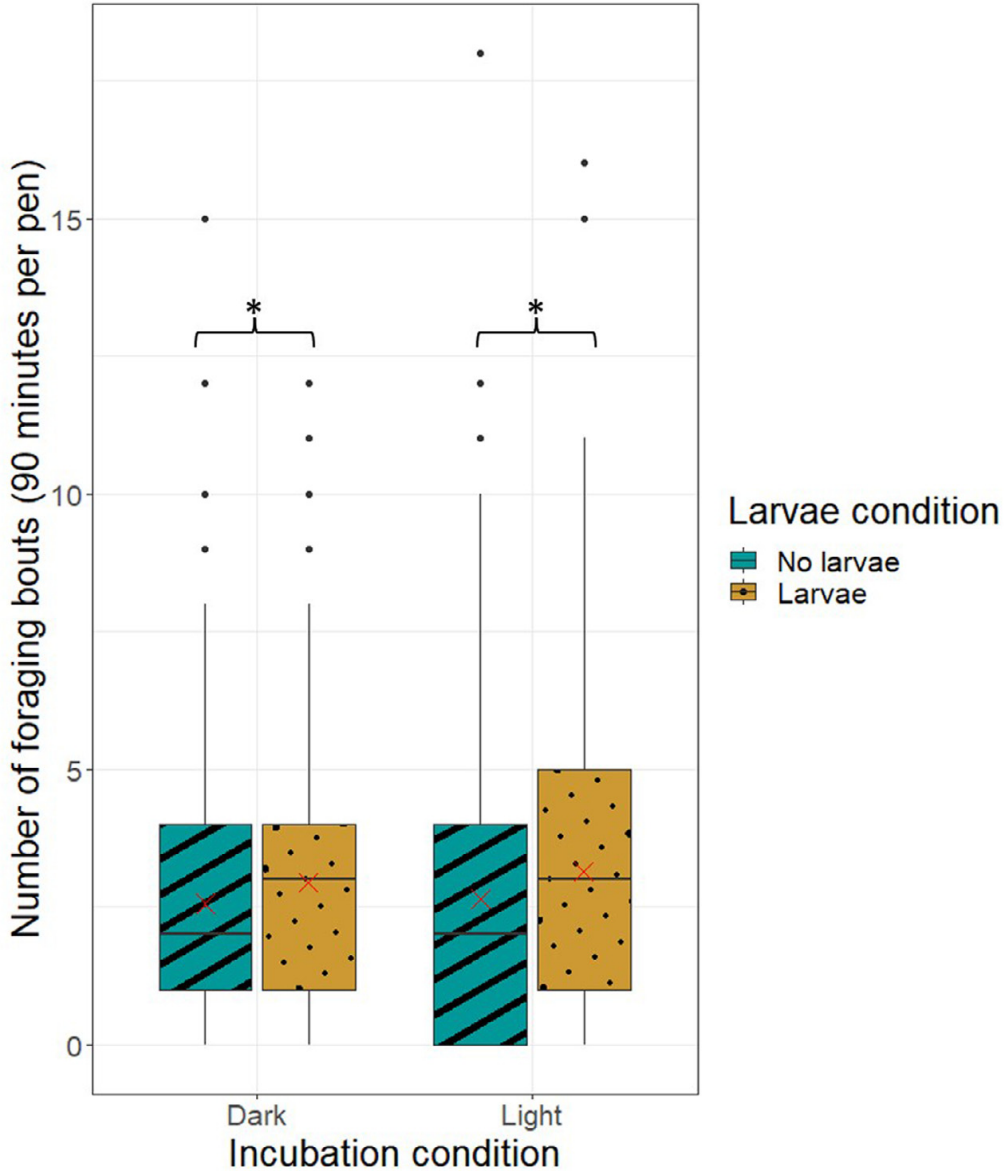
Boxplot of the number of foraging bouts per treatment per total 90-min observation time per pen, during continuous sampling of laying hen pullets. Observations were performed at 1, 4, and 7 wk of age (30 min per pen per age). The X in the box represents the mean.

*Foraging Behavior.* Pullets that received larvae performed more foraging bouts than the pullets that did not receive larvae (RR 1.19, 95% CI 1.05–1.35; *P* = 0.008, Figure 4). In contrast, light during incubation did not affect the number of foraging bouts (RR 1.05, 95% CI 0.92–1.19; *P* = 0.47). In addition, no effects were found of lighted incubation (RR 1.01, CI: 0.90–1.14) or larvae provisioning (RR 1.08, CI: 0.96–1.22) on total foraging duration.

*Batch Effects*. Batch effects were found in the NO test, FP, and Plumage Condition scores. Though batch effects are outside the scope of this study, these results are available in the supplementary data as a contribution to the transparency and reproducibility of animal experiments.

## DISCUSSION

This study investigated the effects of a green LED light-dark cycle throughout incubation and BSFL provisioning during rearing as interventions to reduce fearfulness and the development of FP throughout the rearing phase of laying hens. Neither lighted incubation nor larvae provisioning seemed to affect these behaviors. Larvae enrichment did slightly increase foraging frequency, but not foraging duration.

### Fearfulness and Recovery After a Stressor

No effects of lighted incubation or larvae provisioning were found on the latency to approach a NO or a human. The overall latencies to approach were similar to those found in previous studies (de Haas et al., 2013; van der Eijk et al., 2018). In commercial practice, a reduced fear of humans could improve the birds’ responses during day-to-day interactions with the farmer, for example during inspection rounds. In addition, increased fear of humans in pullets could be a predictor for feather damage in these same birds when adult (De Haas et al., 2014b). In the current experiment, we did not find evidence that light during incubation and BSFL provisioning during rearing can be used as a strategy to reduce fearfulness in commercial practice. In addition, these treatments did not affect the behavioral recovery after vaccination. Preening behavior increased immediately after vaccination in all treatment groups. Possibly, the pullets were motivated to preen excessively, that is, to bring their plumage back in order, after being handled by the vaccinators, although this behavior was not observed after pullets were caught and handled for other tests by the same people. Preening has been postulated to serve as coping or displacement behavior that alleviates stress in wild birds after a stressful event (Delius, 1988; Henson et al., 2012). This so-called displacement preening has also been associated with feather-damaging behavior in parrots (van Zeeland et al., 2009) and frustration in laying hens (Zimmerman et al., 2000; Olsson et al., 2002). Our finding suggests that the vaccination was indeed experienced as stressful for the pullets in this study. This finding contributes to the ambivalent interpretation of preening, that is, it could indicate either stress or comfort (Savory and Kostal, 2006). The interpretation of preening should therefore be context-dependent.

### Feather Pecking and Plumage Score

Lighted incubation and larvae provisioning did not affect FP or plumage score. The pullets in our study rarely showed SFP. Although this damaging behavior usually develops at the onset of egg-laying (Rodenburg et al., 2013), some studies have observed it during the rearing phase in commercial flocks (De Haas et al., 2014a). In another study on lighted incubation in laying hens, green light seemed to increase GFP but decrease SFP and aggressive FP at 24 and 32 wk of age (Özkan et al., 2022). The green light-incubated hens also had a better plumage score at 40 wk compared with white light- and dark-incubated hens. Possibly, effects of the interventions on the pullets in the present study will emerge at a later stage. To test this, a follow-up experiment studying the same hens from 19 until 70 wk of age is currently ongoing (Plante-Ajah et al., unpublished data). Another recommendation is to repeat the experiment with a longer photoperiod during incubation. This might enhance the contrast between treatment and control, such as the 16L:8D study performed by Özkan et al. (2022), or limit light exposure to short episodes during the last phase of incubation to better resemble natural conditions.

### Foraging Behavior

Larvae-enriched pullets initiated more foraging bouts than nonenriched pullets, but total foraging duration was not affected by larvae provisioning. These results imply that foraging enrichment causes more frequent, but shorter moments of foraging. Despite the known association between the provision of sufficient foraging opportunities and reduced FP, the increased number of foraging bouts was not related to lower levels of FP in this study.

This experiment is the first to report on the effects of provisioning BSFL as enrichment throughout the rearing phase of laying hens. Existing literature has been clear about the positive effects of enrichment on the reduction of fearfulness and FP, and the promotion of positive behaviors such as foraging (reviewed by Campbell et al., 2019). One potential reason we did not find strong effects of foraging enrichment could be that not all pullets were eating the larvae. A recent study reported large individual variation in larvae consumption by laying hens (Tahamtani et al., 2021). This finding indicates that the larvae dispensers in the present study might not have provided enrichment to each individual pullet. Competition for, or lack of interest in, the enrichment provided are risks associated with providing enrichment at a group level. In a future experiment, it would be interesting to link individual interaction with enrichment objects to outcome parameters in individual tests. Another important reason we did not find convincing evidence might be related to the enriched environment in which all pullets in the present study were reared. The surface area per bird was relatively large, and pullets were exposed to daylight in the pens.

In addition to enriched housing, the pullets were being handled frequently from hatching onwards due to the large number of measurements and behavior tests performed. Previous studies have shown that handling decreased fear of humans in ISA Brown chicks (Jones, 1994, 1995). The latencies to approach an object or human in our experiment were similar to those in previously mentioned studies (de Haas et al., 2013; van der Eijk et al., 2018), studies that also involved multiple tests and handling. Furthermore, the classical music that was played 24/7 to avoid startle responses by sudden noises in the facility could have reduced stress (Davila et al., 2011). All these factors might be considered enrichment and may have reduced stress responses, fearfulness, and FP to a level that masked the potential effects of the BSFL provisioning. These husbandry aspects might also explain the lack of contrast in fearfulness between the light- and dark-incubated pullets.

Larvae provisioning might still be an effective measure to promote foraging behavior, and perhaps reduce FP in later life. In a farmer survey conducted in 2021, farmers agreed that environmental enrichment is an important welfare-improving lever in poultry production (Kliphuis et al., unpublished data). Given the limited beneficial effects of larvae as enrichment in the present study, ethical and environmental considerations need to be addressed before applying larvae enrichment on a commercial scale. First, larvae should be reared in a way that does not negatively impact their own welfare (Lambert et al., 2021; Barrett et al., 2023). Second, assuming that large-scale insect production is ethically accepted, it should also be sustainable; ideally, insect farms should use food waste as a rearing substrate (Gamborg et al., 2018). This is currently being hampered by legislation for food-safety reasons (Lalander and Vinnerås, 2022). Third, the use of larvae dispensers as done in this study is not feasible on-farm, since filling the dispensers is laborious. A more feasible option that has been shown to increase foraging is manual or automatic scattering of larvae through the litter area. Given the issues to consider when using insects as enrichment, other types of foraging enrichment may be more easily applicable on-farm. For example, providing alfalfa bales and pecking blocks have been shown to reduce FP on commercial rearing farms (Zepp et al., 2018). The use of enrichment in low-input and organic production systems becomes even more important when hens need to be kept indoors as a strategy to reduce the spread of avian influenza—a current welfare problem of substantial proportions worldwide.

## CONCLUSIONS

This study did not show any significant effect of exposure to a green LED light-dark cycle throughout incubation or larvae provisioning on fearfulness, early FP, or plumage score in laying hen pullets. The only significant treatment effect was an increased number of foraging bouts when pullets were provided with larvae. Despite the lack of effects in the current study and given the body of literature that suggests a positive impact of both lighted incubation and enrichment during rearing on behavioral and physiological traits, additional research is recommended to further elucidate the full potential of these strategies to improve laying hen welfare.

## ACKNOWLEDGMENTS

This project received funding from the European Union's Horizon 2020 Research and Innovation Programme under grant agreement N°816172. The authors would like to thank Inge van den Burg, Casper van Eekelen, Dylan Geerman, Margaux Laurent, Elyse van Leeuwen, Lotte Pluim, Antoine Prunier, Arjen van Putten, Lisa Veldkamp, and Jary Weerheijm for their help with the data collection; Jan van den Broek for additional statistical support; Marcel Heetkamp for the incubation process; Freek Weites and Marc Kranenburg for taking care of the pullets; and Mieke Matthijs and Thijs Manders for providing veterinary care.

## DISCLOSURES

T. B. Rodenburg reports financial support was provided by Horizon 2020 Research and Innovation Programme. The remaining authors declare no conflicts of interest.

## Appendix Supplementary materials

Image, application 1

Image, application 2

Image, application 3

## Footnotes

Supplementary material associated with this article can be found in the online version at doi:10.1016/j.psj.2023.102801.
