# Supplementary figures and images for: Early-life interventions to prevent feather pecking and reduce fearfulness in laying hens
Source: Poult Sci. 2023 May 24;102(8):102801. doi: 10.1016/j.psj.2023.102801 (PMC10404761; doi:10.1016/j.psj.2023.102801)

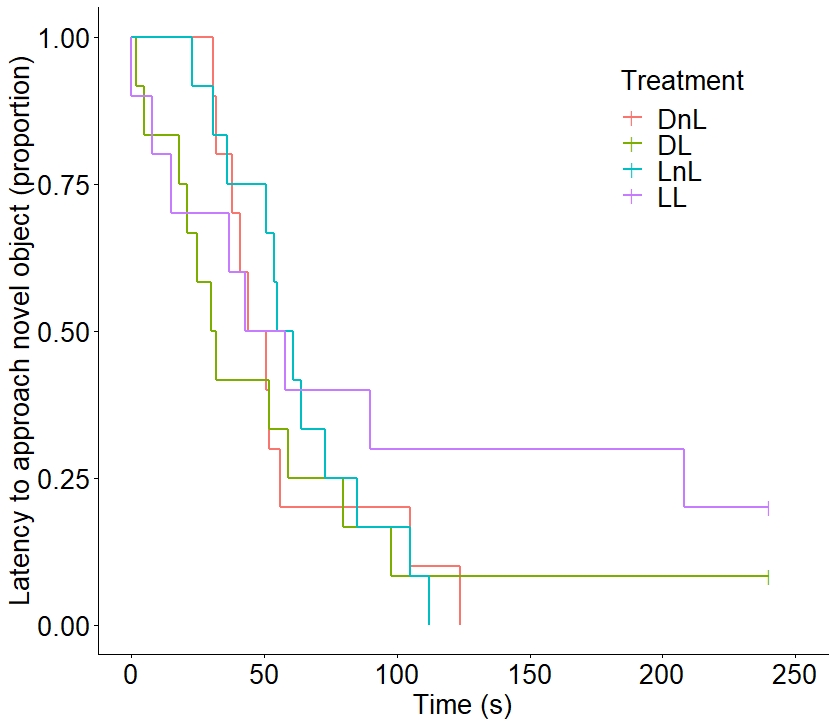

Supplement: Supplementary file 1 [file mmc1.zip › mmc1.jpeg]

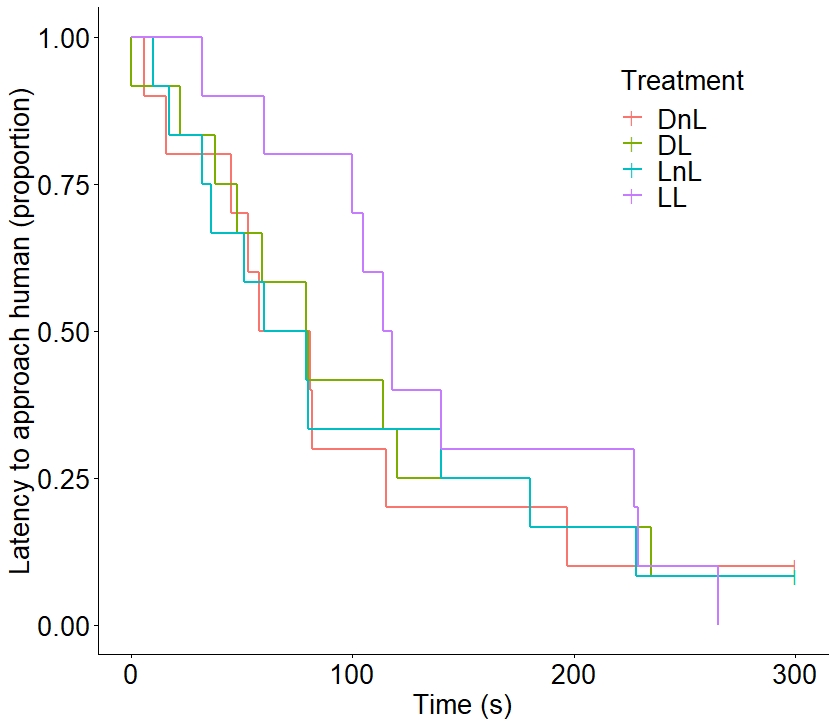

Supplement: Supplementary file 2 [file mmc2.zip › mmc2.jpeg]

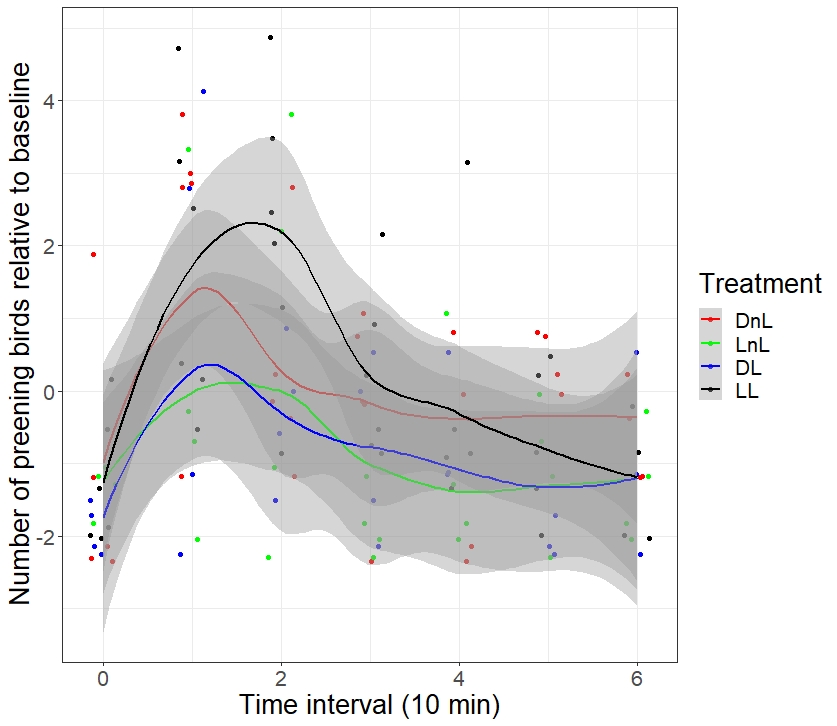

Supplement: Supplementary file 3 [file mmc3.zip › mmc3.jpeg]

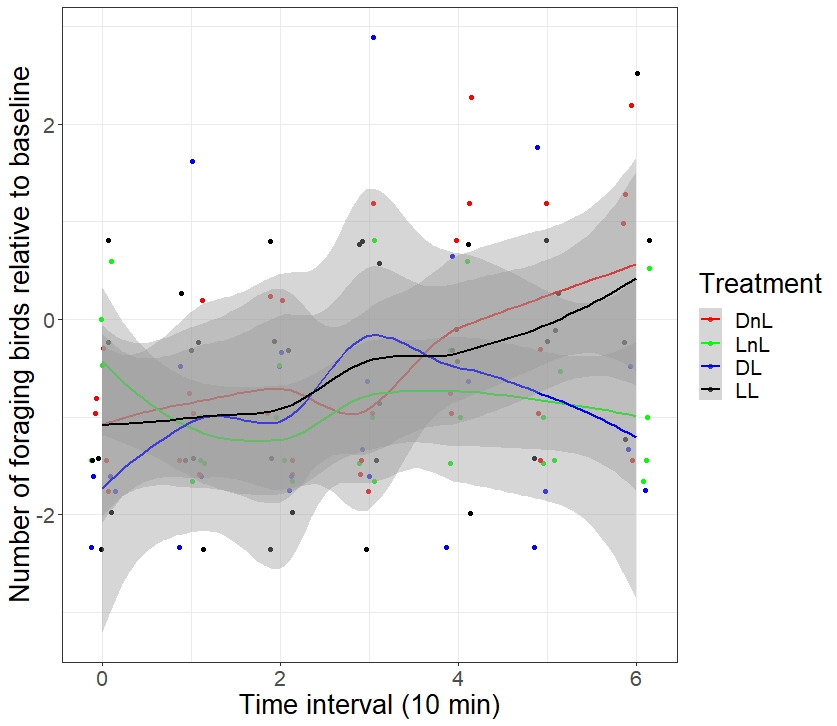

Supplement: Supplementary file 3 [file mmc3.zip › mmc6.jpeg]

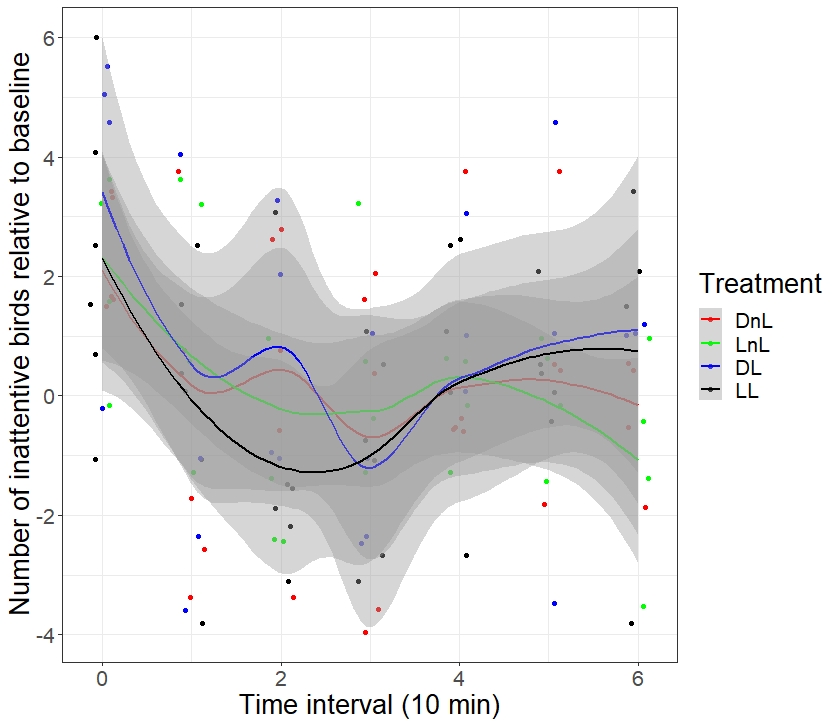

Supplement: Supplementary file 3 [file mmc3.zip › mmc4.jpeg]

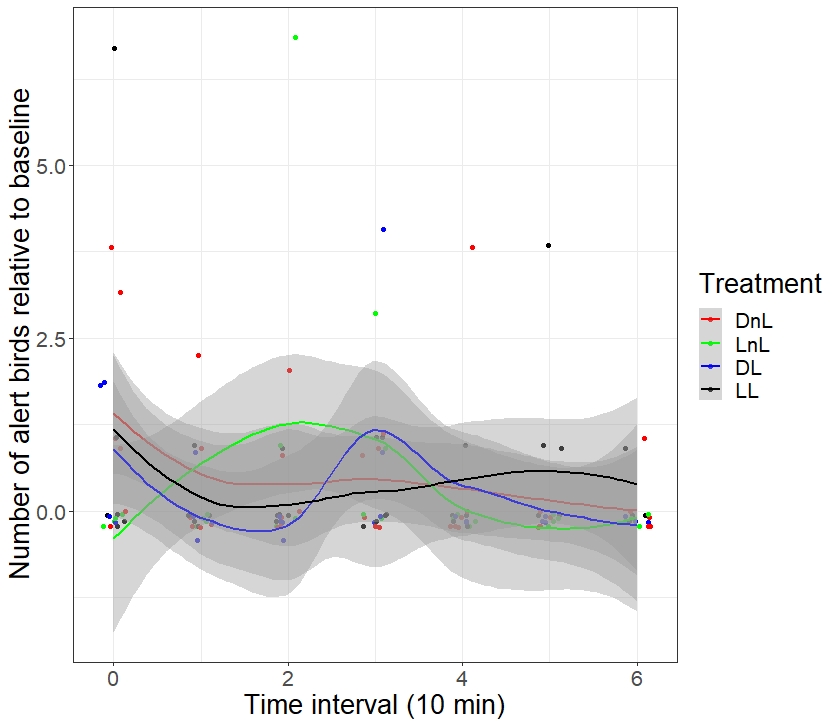

Supplement: Supplementary file 3 [file mmc3.zip › mmc5.jpeg]
