# Supplementary material for: Early-life interventions to prevent feather pecking and reduce fearfulness in laying hens
Source: Poult Sci. 2023 May 24;102(8):102801. doi: 10.1016/j.psj.2023.102801 (PMC10404761; doi:10.1016/j.psj.2023.102801)
Supplement: Supplementary file 3 [file mmc3.zip › supp. Batch effects.docx]

## Batch effects - results

***Novel object test.*** Pullets from batch 2 approached the novel object 11.40 times slower than those from batch 1 (HR, 95% CI 6.26-20.75; p<0.0001). The mean latency to approach in batch 1 was 62.40 ± 12.05 compared with 224.7 ± 0.01 in batch 2.

***Feather pecking.*** 0.34 times fewer GFPs were observed in batch 2 compared with batch 1 (RR, 95% CI 0.27-0.44; p<0.0001). The mean number of GFP was 51.14 ± 5.77 in batch 1 compared with 16.67 ± 1.19 in batch 2.

***Plumage condition***. Pullets in batch 2 had a lower cumulative score than pullets in batch 1, indicating a better plumage condition (OR 2.57, 95% CI 1.43-4.70).

## Batch effects - discussion

The differences in outcome parameters between batches were remarkable. They started to show early in the study, during the novel object test performed at 6 days of age, indicating that these differences may develop early on. In batch 1, two pullets were euthanized because of severe eye infections. The peat litter was believed to be the cause of this and, following veterinary advice, we changed the bedding material to wood shavings when the pullets were 8 weeks old. We kept wood shavings as litter material throughout batch 2. A recent study showed that litter type can affect chick behavior (Skånberg et al., 2021), although the behavior traits studied were different from the ones in the present study. Another cause for the batch effect might be the age of the parent stock (43 weeks in batch 1 and 34 weeks in batch 2); however, parent stock age did not affect fear-related behavior and FP in a previous study in commercial flocks (de Haas et al., 2014).

The 2 batches were not observed by the exact same individuals, which could explain part, but not all variability, as the observers were balanced over treatments. The seasonal differences between batch 1 (February to June) and batch 2 (May to September), e.g., sunlight might have entered the poultry house at a different angle, may also have affected behavior. We did not measure any extreme temperatures on testing days and treatments were balanced over pen location.
